# Using AI and Gd-EOB-DTPA-enhanced MR imaging to assess liver function, comparing the MELIF score with the ALBI score

**DOI:** 10.1038/s41598-023-39954-1

**Published:** 2023-08-12

**Authors:** Carolina Río Bártulos, Karin Senk, Ragnar Bade, Mona Schumacher, Nico Kaiser, Jan Plath, Mathis Planert, Christian Stroszczynski, Jan Woetzel, Philipp Wiggermann

**Affiliations:** 1 Institut Für Röntgendiagnostik Und Nuklearmedizin, Städtisches Klinikum Braunschweig gGmbH, 38126 Braunschweig, Germany; 2 Institut Für Röntgendiagnostik, Universitätsklinikum Regensburg, 93053 Regensburg, Germany; 3 MeVis Medical Solutions AG, 28359 Bremen, Germany

**Keywords:** Diagnostic markers, Hepatology, Liver diseases, Diagnostic markers

## Abstract

Monitoring disease progression is particularly important for determining the optimal treatment strategy in patients with liver disease. Especially for patients with diseases that have a reversible course, there is a lack of suitable tools for monitoring liver function. The development and establishment of such tools is very important, especially in view of the expected increase in such diseases in the future. Image-based liver function parameters, such as the T1 relaxometry-based MELIF score, are ideally suited for this purpose. The determination of this new liver function score is fully automated by software developed with AI technology. In this study, the MELIF score is compared with the widely used ALBI score. The ALBI score was used as a benchmark, as it has been shown to better capture the progression of less severe liver disease than the MELD and Child‒Pugh scores. In this study, we retrospectively determined the ALBI and MELIF scores for 150 patients, compared these scores with the corresponding MELD and Child‒Pugh scores (Pearson correlation), and examined the ability of these scores to discriminate between good and impaired liver function (AUC: MELIF 0.8; ALBI 0.77) and to distinguish between patients with and without cirrhosis (AUC: MELIF 0.83, ALBI 0.79). The MELIF score performed more favourably than the ALBI score and may also be suitable for monitoring mild disease progression. Thus, the MELIF score is promising for closing the gap in the available early-stage liver disease monitoring tools (i.e., identification of liver disease at a potentially reversible stage before chronic liver disease develops).

## Introduction

Due to the global prevalence in nonalcoholic fatty liver disease (NAFLD), estimated to be 24% and rising, an increase in chronic liver disease (CLD) is expected; CLD often progresses to cirrhosis and may develop into hepatocellular carcinoma (HCC)^1–3^. Therefore, for patients with liver disease, determining and monitoring liver function is crucial.

In clinical practice, liver function and the severity of liver damage are usually determined on the basis of blood values, e.g., bilirubin, alkaline phosphatase, aspartate transaminase, glutamyl transferase, alanine transaminase, albumin, and prothrombin time^4,5^. Consequently, blood parameters are an integral part of the various scoring systems developed to determine the severity of liver disease and are often used as surrogate parameters for liver function^6^. The Child‒Pugh score is one of the oldest scoring systems in use. This scoring system was developed to determine which cirrhotic patients would benefit from surgical intervention for portal vein decompression^7,8^. Currently, it is used in the form modified by Pugh to classify patients with cirrhosis into different stages according to the severity of their symptoms^9^. It divides patients into Child‒Pugh stages A-C, where A represents mild cirrhosis and C represents decompensated cirrhosis, and is used to predict prognosis and make decisions about the type of therapy or the need for liver transplantation. This scoring system includes the following parameters: serum albumin, serum bilirubin, International Normalized Ratio (INR) and the subjective parameters degree of ascites and encephalopathy. In particular, subjective parameters, which can be influenced by medication, have been criticized, leading to the introduction of the model for end-stage liver disease (MELD) score^10^. The MELD score is used for the allocation of donor livers and has predictive value for the severity of liver disease and mortality risk^11^. It is based on the blood values of serum bilirubin, serum creatinine and INR^10,12^. A more recent scoring system is the ALBI score/grade, which is based on the blood values of albumin and bilirubin and was originally developed to assess liver function in HCC patients^13^. This scoring system has emerged as a prognostic indicator for all HCC treatments and is used to stratify patients with HCC finely than the CP score or MELD scoring systems could^14,15^. It correlates with survival, time to relapse, and tolerability of locoregional treatments for HCC^16^. Furthermore, it has been shown to be a reliable alternative for assessing the extent of liver impairment and is sensitive to minor deteriorations in liver function. This property makes it a good candidate for determining liver function at an early stage of disease. Similar to the blood-based scoring systems previously mentioned, it is also used beyond its original scope to assess liver function^14^. However, there is a need for tools that can reliably determine liver function at an early stage of disease and that can be used to detect disease progression, preferably before end-stage liver disease is reached. Nevertheless, these blood-based scoring systems have the advantage of simplicity in clinical practice, while also representing static tests. In addition, there is evidence that the ALBI score is not liver specific. For example, it has also been studied in diseases affecting organs other than the liver, such as cardiac and pancreatic diseases, in which it can also serve as a prognostic factor, as both bilirubin and albumin metabolism may be impaired in other chronic diseases; furthermore, many diseases are not exclusively confined to one organ, and the liver has a central role in whole body metabolism^14,17–20^.

An alternative to these blood-based liver function values is the determination of liver function by using radiological images, which is a more direct method. For this purpose, magnetic resonance imaging (MRI) seems to be suitable, especially since hepatocyte-specific contrast agents, such as gadoxetic acid (Gd-EOB-DTPA), are available. Here, the T1 reduction rate (rrT1) calculated from T1 maps before and after contrast administration has been shown to be a reliable prognostic and diagnostic liver function score for liver disease^21–24^. Although this value is promising, it has been used only in research, in part due to its requirement for time-consuming manual calculations. With the help of artificial intelligence (AI) and deep learning, it has become possible to develop software that fully automatically calculates a liver function score based on the rrT1, the so-called MELIF score. The MELIF score considers the rrT1 value of the entire liver instead of the values of individual regions of interest and incorporates other patient-specific factors such as height, weight and liver volume^25^. This provides a tool that can automatically determine regional liver function from imaging data, and in a first retrospective analysis, the MELIF score was shown to correlate better with the MELD score than the rrT1 score of the whole liver^25^.

In this retrospective study, a side-by-side comparison of the MELIF score with the ALBI score was performed to better assess the diagnostic potential of the MELIF score. Therefore, the correlation of the MELIF and ALBI scores with the CP and MELD scores was first investigated. Furthermore, in a more in-depth analysis, a direct comparison between the ALBI and MELIF scores was made by grouping patients into good and poor liver function groups according to the MELD score and, in a second approach, into groups of patients with and without cirrhosis. Finally, the diagnostic ability of MELIF score to distinguish the different ALBI grades was investigated.

## Results

### Characteristics of the study population

**Figure 1:**
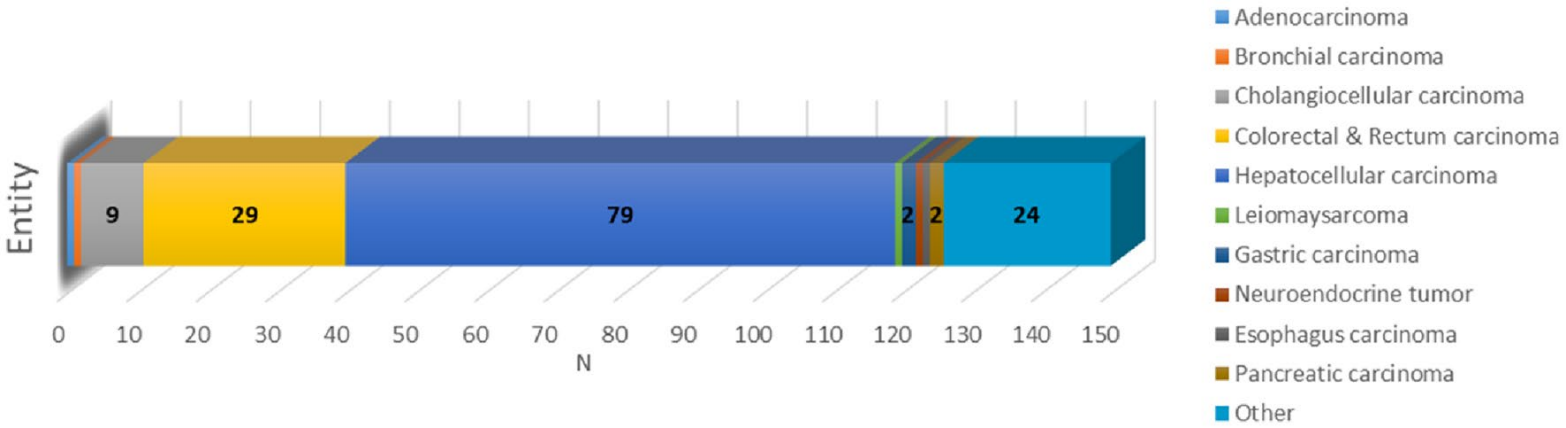
Overview of the patient population. Malignant lesions are presented, while patients with benign lesions or other liver diseases are grouped under Other. Numbers are given only for groups with more than one patient. N = number.

The present study included 150 patients with a mean age of 62 years, 82% of whom were male (Table 1). There were 126 patients with malignant liver lesions, including 79 patients with HCC and 29 patients with colorectal and rectal carcinoma (Fig. 1). The 24 patients without malignant liver lesions included patients with benign liver lesions (e.g., cysts, haemangiomas, or adenomas) as well as patients with liver disease (NAFLD, hepatitis, or cirrhosis). Overall, 86 patients had cirrhosis, most of whom were Child‒Pugh stage A or B (Table 1). One patient underwent liver transplantation, and 53 patients underwent partial liver resection.

**Table 1:** Characteristics of the study population.

	All (150)
Sex (M/F)	123/27 (82/18%)
Age (years)	62 (± 10)
Height (m)	1,7 (± 0,08)
Weight (kg)	83 (± 16)
Liver volume (ml)	1547 (± 391)
MELD	9 (7–11)
MELIF	50 (± 11)
Albumin (g/dl)	3.3 (± 0.63)
ALBI score	− 2.0 (± 0.60)
ALBI grade 1	33 (22%)
ALBI grade 2	95 (63%)
ALBI grade 3	22 (15%)
Cirrhosis (yes)	86 (57%)
Child Pugh	
A	45 (30%)
B	38 (25%)
C	3 (2%)

Data are presented as N (%) for categorical variables and mean (± standard deviation) or median (interquartile range) for continuous variables. MELD = model for end-stage liver disease, M = male, F = female.

### Correlation of the MELIF score with the ALBI, MELD and CP scores

Liver function values were calculated for all 150 patients. The median MELD score was 9 (IQR = 7–11), the mean MELIF score was 50 (± 11), and the mean ALBI score was − 2.0 (± 0,6) (see Table 1). The Pearson correlation was chosen to evaluate the relationship between the different scores. The result of the correlation is shown in Table 2. All scores showed a strong and significant correlation with each other (r =  > 0.5). The MELIF score correlated more strongly with the MELD (r = − 0.61) and CP (r = − 0.66) scores than the ALBI score correlated with these scores (r = 0.54 and 0.63, respectively). The correlation between the MELIF and ALBI scores was r = − 0.65.

**Table 2:** Pearson correlation of liver function scores.

	ALBI	MELD	CP
MELIF	− 0.65 (CI − 0.73 to − 0.55) *	− 0.61 (CI − 0.7 to − 0.5) *	− 0.66 (CI − 0.74 to − 0.56) *
	ALBI	0.54 (CI 0.42 to 0.65) *	0,63 (CI 0.52 to 0.72) *
		MELD	0.56 (CI 0.44 to 0.66) *

MELD = model for end− stage liver disease, CP = Child‒Pugh, CI = 95% confidence interval, * = two tailed *p* < 0.0001.

### Comparison of ALBI and MELIF

To obtain a better idea of whether the new MELIF score can compete with the established scores, the ALBI score was used as a benchmark. For the direct comparison of the MELIF and ALBI scores, two approaches were chosen. The first approach examined the ability of the two scores to discriminate between good and impaired liver function by dividing the 150 patients into two groups based on their MELD score, with a MELD score ≤ 10 representing good liver function and a score ≥ 11 representing impaired liver function. Overall, 103 patients (69%) had a MELD score ≤ 10, with a mean MELIF score of 54 (± 9.8) and a mean ALBI score of − 2.2 (± 0.52); 47 patients (31%) had a MELD score ≥ 11, with a mean MELIF score of 42 (± 10) and an ALBI score of − 1.7 (± 0.58) (Table 3).

**Table 3:** Mean MELIF and ALBI scores for the MELD ≤ 10, MELD ≥ 11, no cirrhosis, and cirrhosis groups. The standard deviation or the number (N) is given in parentheses.

	MELIF	ALBI
MELD ≤ 10 (103)	54 (± 9.8)	− 2.2 (± 0.52)
MELD ≥ 11 (47)	42 (± 10)	− 1.7 (± 0.58)
No cirrhosis (64)	58 (± 8.9)	− 2.4 (± 0.48)
Cirrhosis (86)	45 (± 10)	− 1.8 (± 0.57)

The second approach was to show whether the scores could distinguish between patients with cirrhosis and those without cirrhosis. For this purpose, the patients were also divided into two groups: those with cirrhosis, 57% (N = 86) and those without cirrhosis, 43% (N = 64). The mean MELIF score of the group without cirrhosis was 58 (± 8.9), and the mean ALBI score was − 2.4 (± 0.48); in the cirrhosis group, the mean MELIF score was 45 (± 10), and the mean ALBI score was − 1.8 (± 0.57) (see Table 3).

**Figure 2:**
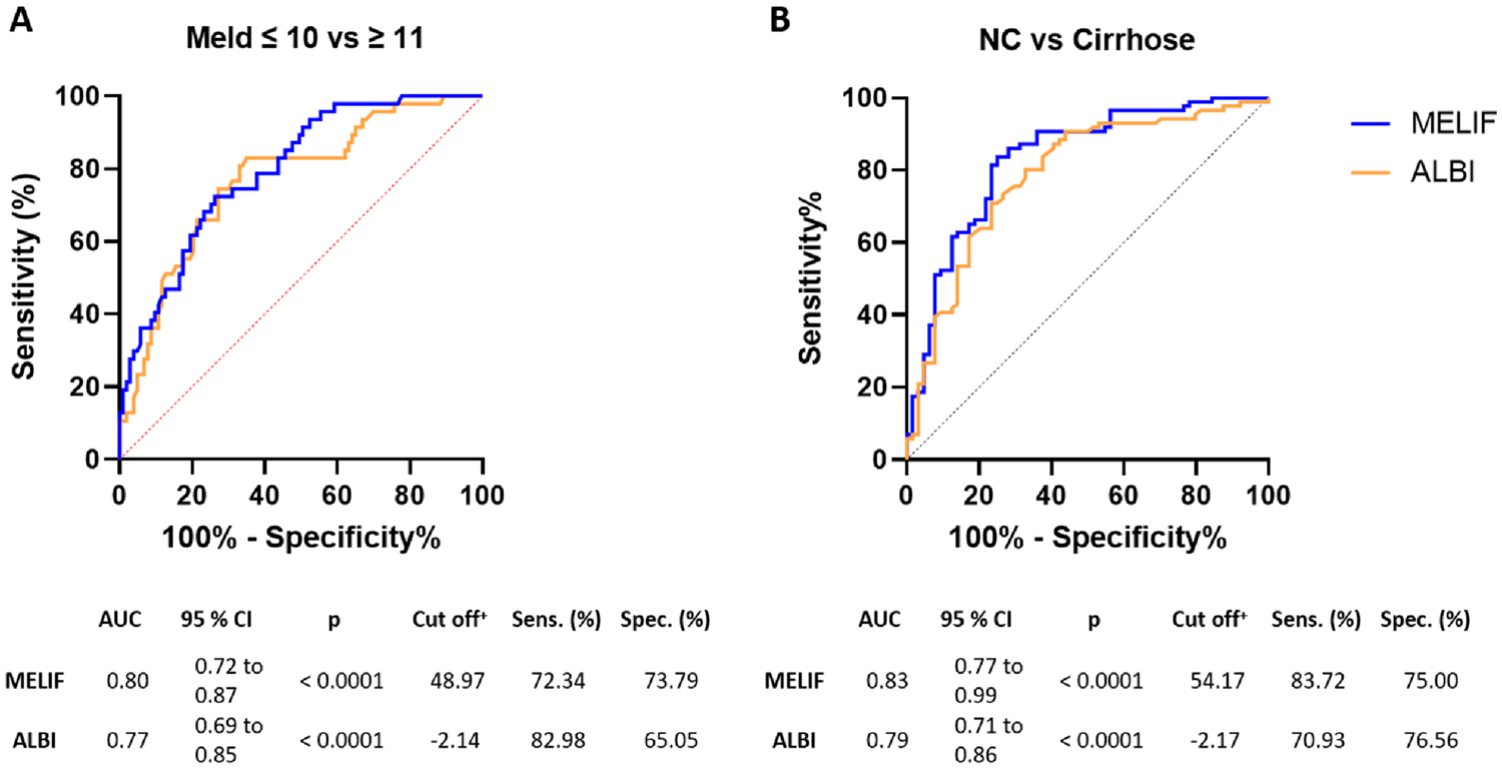
ROC curve predicting (**A**) impaired liver function or (**B**) liver cirrhosis using the MELIF (blue line) or the ALBI score (orange line). The respective AUC analysis together with the calculated cut-off value and the corresponding sensitivity and specificity are shown below the graph. NC = no cirrhosis, ^+^  = calculated using the Youden index^26^, CI = confidence interval, Sens. = sensitivity, Spec. = specificity.

The area under the receiver operating curve (AUC) was used to compare the model accuracy of the MELIF and ALBI scores. Figure 2 shows the results of the analysis. The MELIF score had a slightly higher AUC score than the ALBI score, both in distinguishing between good and impaired liver function and between patients with and without cirrhosis. The AUC value for discriminating between good and impaired liver function for the MELIF score was 0.80, which demonstrated good accuracy, while the ALBI score for this data set had a fair accuracy of 0.77. Similarly, the accuracy of the MELIF score in distinguishing between patients with and without cirrhosis was 0.83 (good), whereas that of the ALBI score was 0.79 (fair). By means of the Youden index, a cut-off value was determined (Fig. 2), and the determined sensitivities and specificities were comparable.

### The interconnection of the MELIF score to the ALBI grade groups

**Figure 3:**
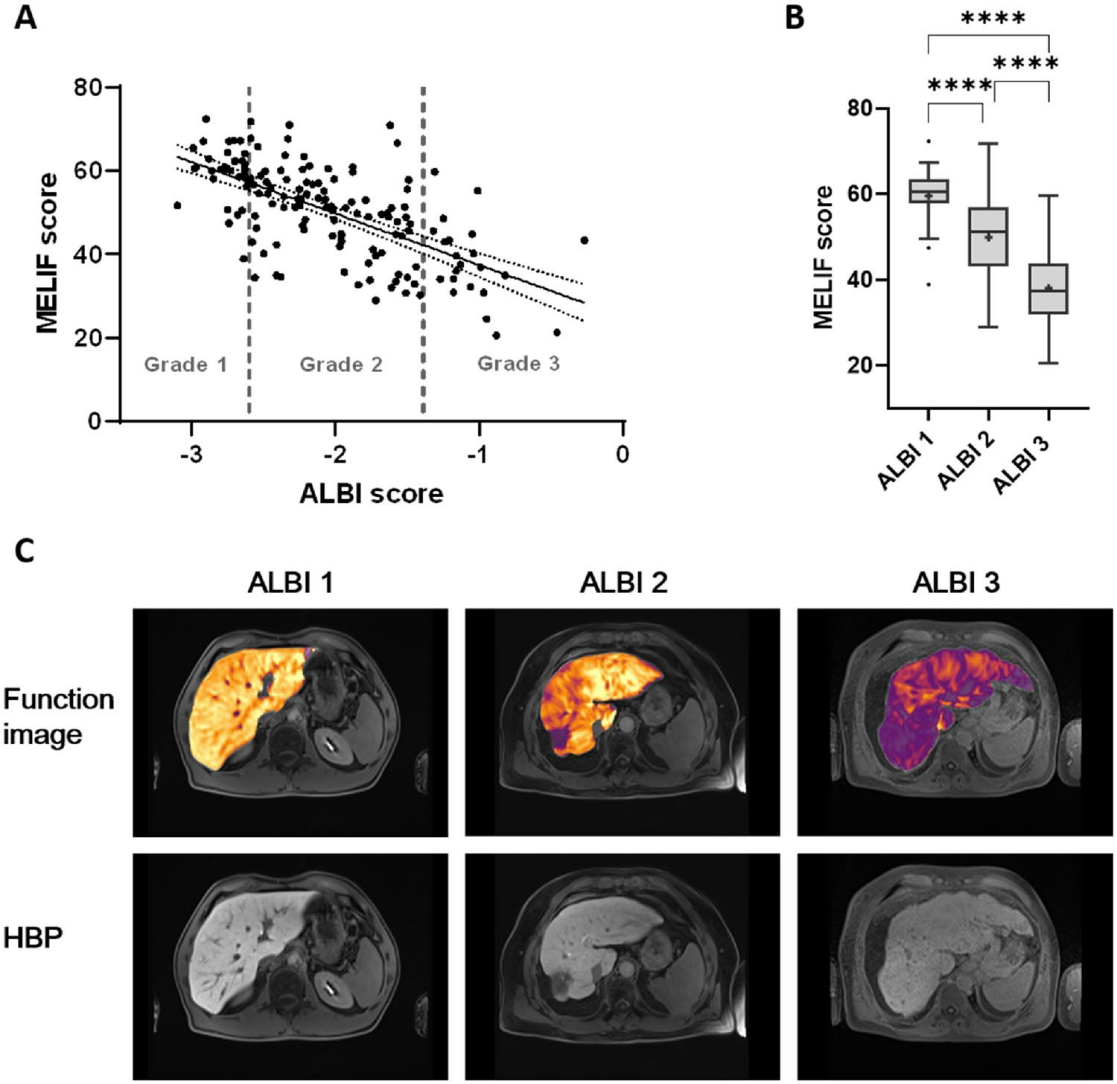
MELIF score versus ALBI grade groups. (**A**) Correlation matrix between the MELIF and ALBI scores. In the graph, the ALBI grades are drawn in grey. A simple linear regression with a confidence interval of 95% is shown as a straight line with dotted lines. (**B**) Boxplots of the MELIF score for patients assigned to ALBI grades 1–3 and pairwise comparison calculated with one-way ANOVA (**** ≤ 0.001). Tukey boxplots are shown, and + represents the mean value. (**C**) Example images of the MELIF score representation by the software for ALBI grades 1–3. ALBI 1 depicts a liver with a MELIF score of 64, a MELD score of 6, and an ALBI score of − 2.63. ALBI 2 depicts a liver with a MELIF score of 51, a MELD score of 12, and an ALBI score of − 2.06. ALBI 3 depicts a liver with a MELIF score of 34, a MELD score of 17, and an ALBI score of − 1.08. HBP = T1-weighted image after contrast administration at the hepatobiliary phase.

Finally, to further investigate the relationship between the MELIF and ALBI scores, patients were divided into three groups based on their ALBI score. ALBI grade 1 represented patients with good liver function, while ALBI grades 2 and 3 represented increasingly impaired liver function. As shown in Table 4, 22% (N = 33) of patients belonged to the ALBI grade 1 group, 63% (N = 95) of patients belonged to the ALBI grade 2 group, and 15% (N = 22) of patients belonged to the ALBI grade 3 group. The mean age was comparable among all three groups, as was the distribution of male and female patients. Figure 3A shows the correlation matrix between the MELIF and ALBI scores, as well as the ALBI grade groups.

**Table 4:** Study population characteristics by ALBI grade groups.

	ALBI grade 1 (33)	ALBI grade 2 (95)	ALBI grade 3 (22)
Sex (M/F)	26/7 (79/21%)	82/13 (86/14%)	15/7 (68/32%)
Age (years)	61 (± 11)	63 (± 11)	64 (± 8.4)
MELIF	60 (± 6,7)	50 (± 10)	38 (± 9.8)
MELD	7 (6–8)	9 (7–11)	12 (8.8–14)
ALBI score	− 2.8 (± 0,13)	− 2 (± 0.36)	− 1 (± 0.26)
Albumin (g/dl)	4 (± 0.25)	3.3 (± 0.42)	2.3 (± 0.3)
Child Pugh	6 (18%)	61 (64%)	19 (86%)
A	6	39	–
B	–	22	16
C	–	–	3

Data are presented as N (%) for categorical variables and mean (± standard deviation) or median (interquartile range) for continuous variables. MELD = model for end-stage liver disease, M = male, F = female.

The mean MELIF score of the ALBI grade 1 group was 60 (± 6.7), that of ALBI grade 2 was 50 (± 10), and that of ALBI grade 3 was 38 (± 9.8). The pairwise comparison of the means showed that they were significantly different (Fig. 3B). An example of how the software displays the MELIF score for the different ALBI grades is shown in Fig. 3C, in which yellow represents good liver function, and purple represents impaired liver function.

**Figure 4:**
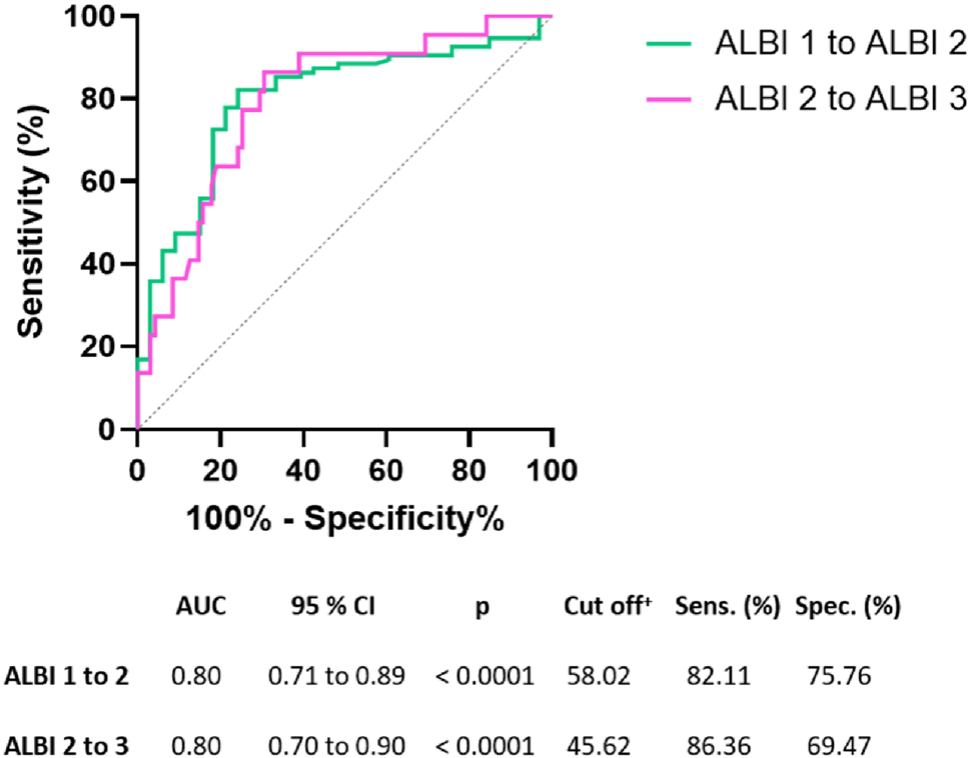
ROC curve of the MELIF score to distinguish patients with ALBI grade 1–2 (green line) or ALBI grade 2–3 (magenta line) with the corresponding AUC analysis including the calculated cut-off value and the corresponding sensitivity (Sens.) and specificity (Spec.). CI = confidence interval; ^+^  = calculated using the Youden index^26^.

In addition, an AUC analysis was performed to determine the extent to which the MELIF score could distinguish patients in the ALBI grade 1 from the ALBI grade 2 group or the ALBI grade 2 from the ALBI grade 3 group. Figure 4 shows the ROC and AUC analyses. The AUC was 0.8 in both cases. A cut-off value was determined using the Youden index, which had a MELIF score of 58.02 to distinguish between patients with ALBI grades 1 and 2, with a high sensitivity of 82.11% and a high specificity of 75.76%. The cut-off value for distinguishing between ALBI grade 2 and ALBI grade 3 was a MELIF score of 45.62, with a sensitivity of 86.36% and a specificity of 69.47%.

## Discussion

In this study, the MELIF score was shown to have a strong correlation with global blood-based parameters of liver function, such as the ALBI, the MELD and the Child–Pugh score. The MELIF score was found to discriminate more favourably between patients with good and impaired liver function or with and without cirrhosis when compared directly with the ALBI score.

The use of appropriate stratification, such as scoring systems, has become an essential element in a number of liver diseases for providing prognosis and stage-specific treatment recommendations. One such example is the CP classification in the context of HCC treatment algorithms, such as the Barcelona Clinic Liver Cancer (BCLC) strategy. These scoring systems enable physicians to assess the severity of the disease and determine the most appropriate therapy for the patient. In addition, these scoring systems have been shown to be useful surrogate parameters for assessing liver function and providing prognostic value for the outcome of liver resection^16^. In this context, it is important to assess the liver function of the remnant before surgery to avoid postoperative liver failure (PHLF). These scoring systems are also used in locoregional therapies to assess liver function^27,28^. In addition, the MELD and CP scores are part of the strategies for prognosis prediction and treatment recommendation for HCC, such as the BCLC strategy or that of the Japan Society of Hepatology (JSH). Both of these clinical guidelines have incorporated the ALBI scoring system as an important component in their latest version^29–31^. There is growing evidence in clinical practice that the ALBI score/grade is more appropriate than the MELD or CP score^16,32,33^. Using 2099 data points from HCC patients in a retrospective study, the ALBI grade showed better prognostic performance than that of CP grade in a survival analysis, as well as a better grade distribution^34^. In addition, another retrospective study demonstrated that the ALBI score can predict postoperative short-term outcomes after liver resection and can be used as a preoperative risk assessment tool^35^. Another retrospective study of 473 HCC patients showed that the ALBI score had better predictive value for PHLF than the CP score or the MELD score^36^. Therefore, the ALBI score is particularly well suited for comparison with the newly developed MELIF score. Moreover, both methods are based exclusively on objective parameters.

The MELIF score is based on T1 relaxometry, in which the T1 reduction rate (rrT1) is calculated from parametric maps obtained by the VFA method before and after contrast administration (native and hepatobiliary phase). Compared to the classic rrT1, which is based on manual measurements of individual regions of interest, the MELIF score is calculated fully automatically for the entire liver and takes into account several patient-specific factors such as size, weight and liver volume. In the first retrospective study to investigate this matter, the MELIF score was shown to perform better than the whole liver rrT1 score based on the MELD score as a reference for liver function^25^. In this study, the MELIF score correlated strongly with surrogate parameters of liver function, such as the MELD, CP, and ALBI scores, and it demonstrated the strongest correlation with the ALBI score (r = − 0.65). Compared with the ALBI score, the MELIF score has a stronger correlation with the MELD (r = 0.54 vs. − 0.61) and CP (r = 0.63 vs. − 0.66) scores. The AUC analysis showed that both the MELIF score and the ALBI score were able to discriminate between patients with good and impaired liver function, as defined by the MELD score, as well as between patients with and without cirrhosis. However, the MELIF score had a higher AUC value than the ALBI score (AUC 0.8 versus 0.77 and 0.83 versus 0.79). These results show that the MELIF score is at least as good, if not better, than the ALBI score in determining liver function. Furthermore, the MELIF score was able to discriminate between the different ALBI grade groups. To our knowledge, there are no studies comparing rrT1, which is the basis for the MELIF score, with the ALBI score/grade system. For this reason, a comparison using the existing literature is not straightforward. Nevertheless, some publications have compared other liver function parameters obtained from Gd-EOB-DTPA-enhanced MRI images with the ALBI score. Beer et al. showed a correlation between the ALBI and MELD scores of 0.645 and a correlation between the ALBI and CP scores of 0.674, which is slightly higher than the correlations found here (ALBI score to MELD score, r = 0.54; ALBI score to CP score, r = 0.63). In their study involving patients with chronic liver disease, they compared the ALBI score with various liver function parameters based on signal intensities (SI) and found that these parameters were also able to distinguish between the ALBI grade groups, but the correlation of these parameters with the ALBI score (r between − 0.491 and − 0.529) or the MELD (r between − 0.449 and − 0.462) and CP (r between − 0.432 and − 0.465) scores was weaker than that of the MELIF score in this study^37^. Another study also testing an SI-based parameter, the quantitative liver-spleen contrast ratio, was also able to significantly distinguish between the three ALBI grade groups and showed a correlation of − 0.61 with the ALBI score and r = − 0.35 with the CP score, both of which are lower than the T1 relaxometry-based MELIF score in this study^38^. These weaker correlations may be because T1 relaxometry-based MRI liver function parameters are considered superior to SI-based parameters, as has been pointed out in other studies^6,39^. The cut-off values determined in this study clearly show that there is a need for further studies. For the distinction of good and impaired liver function, the MELIF score was 49; for the distinction of patients with and without cirrhosis, the MELIF score was 54; and for the distinction of ALBI grade 1 to grade 2, the MELIF score was 58, where a higher value indicated better liver function. It is clear that the MELD and CP scores are intended for application in more severely ill patients than the ALBI score. This suggests that the MELIF score, similar to the ALBI score, can also indicate mild disease and thus, similar to the ALBI score, can be used to monitor patients before they reach chronic disease status.

However, the lack of comparisons can be addressed only by future studies. Another limitation of this study was its retrospective nature, although this is very helpful for the initial evaluation of a new score's potential. A further limitation was that only image data from one type of scanner was used; however, this has been shown in numerous studies to have no effect on T1 relaxometry-based scores. In this study, only a few patients with severe liver impairment (Child Pugh C, the worst MELD score was 22) were included because these patients rarely undergo MRI in routine clinical practice, and the MELD and CP scores have been proven useful for monitoring liver function in this group of patients. In addition, the patient population in this study was very heterogeneous, which was perfectly acceptable and negligible for the type of study presented here. Nevertheless, the population should be more homogeneous in future studies analysing the prognostic capabilities of the MELIFs score. Henceforth, prospective studies and trials assessing the MELIF score for its prognostic abilities in the context of liver interventions need to be conducted. Additionally, the use of the MELIF score in the context of locoregional treatments should be investigated to determine whether it is an indicator of overall survival, time to relapse, and treatment tolerability.

In summary, the MELIF score is a novel image-based, fully automatically computable liver function score determined by software using AI technology. It has the ability to determine liver function with spatial resolution, and it performs similarly well or even better than the ALBI score/grade system, which is becoming the new standard in treatment guidelines for liver disease. It remains to be seen how well established the MELIF score will become and whether it will be able to assess liver function and prognosis in early stages of liver disease similarly or even better than the ALBI score.

## Material and methods

### Clinical data collection and study design

This study was performed in accordance with the Declaration of Helsinki and approved by the Institutional Ethics Committee of the University of Regensburg (protocol codes 16-101-0177 and 16-177_1-101; March 5, 2020).

This retrospective study included the records of 150 patients who underwent an MRI examination of the liver with Gd-EOB-DTPA as a liver-specific contrast agent, which was administered as a body-weight-adjusted bolus injection, according to the manufacturer (Primovist©, Bayer Schering Pharma AG, Berlin, Germany). The MRI examinations took place as part of the patients' routine clinical examination. Patients underwent MRI examination for various reasons, including clarification of liver lesions detected by other examinations, a follow-up after treatment of a malignant liver lesion, or monitoring of liver disease. None of the patients had any contraindication to MRI examination or administration of the contrast agent, and all gave their written informed consent. In addition, blood parameters had to be available for each patient to calculate the ALBI, MELD, and CP scores.

### Image acquisition

Imaging was performed using a clinical 3.0-T whole-body system (Magnetom Skyra, Siemens Healthcare, Erlangen, Germany) and a combination of body and spine array coils (18-channel body matrix coil and 32-channel spine matrix coil) for signal reception. In addition to the routine imaging protocol, T1 relaxometry was performed before and approximately 20 min after contrast administration. The B1-corrected variable flip angle (VFA) method was used to generate T1 maps. The VFAs used were 1°, 7°, and 14°, the repetition time (TR) was 5.79 ms, the time to echo (TE) was 2.46 ms and 3.69 ms, and the voxel size was measured as 3.6 mm*2.5 mm*4.8 mm interpolated to 1.3 mm*1.3 mm*3.0 mm. The acquisition was performed in a single breath hold. Attention was given to ensure that the entire liver was represented in the images.

### Calculation of the scores

The MELD score was calculated using Eq. (1). A MELD score of 10 or less indicates normal liver function, while a score of 11 or more indicates impaired liver function.

1 $$MELD \,score=3.78*\mathrm{ln}\,[serum \,bilirubin \,(mg/dL)]+11.2* \mathrm{ln}\,[INR] +9.57*\mathrm{ln}\,[serum \,creatinine \,(mg/dL)]+6.43$$

The CP score was calculated as described by Pugh et al.^9^. For the evaluation performed here, patients who did not have cirrhosis were assigned to the no cirrhosis (NC) group, and all patients who had cirrhosis were assigned to the cirrhosis group, regardless of the score. The presence of cirrhosis was inferred from patient records and biopsy reports.

The ALBI score was calculated according to Eq. (2) proposed by Johnson et al.^13^. The grading was performed according to the proposed classification, where grade 1 represented an ALBI value of less than or equal to − 2.6, grade 2 represented an ALBI value of more than − 2.6 to less than or equal to − 1.39, and grade 3 represented an ALBI value of more than − 1.39. Grade 1 represented good liver function, while grades 2 and 3 represented increasing impairment of liver function.

2 $$ALBI \,score=({\mathrm{log}}_{10}\,bilirubin\, \left[\mu \frac{mol}{L}\right]*0.66)+(albumin\, [g/L]*-0.085)$$

The MELIF score was calculated fully automatically using prototype software, with Eq. (3) describing the previously published calculation methodology^25^. The equation contains patient-specific factors (p) such as height, weight and liver volume. These factors were extracted directly from the image information, and the liver volume calculation was performed by using an integrated segmentation of the liver. Based on AI and deep learning, this software automatically performed segmentation of the liver and performed two registrations. The first consisted of registering the T1 maps before and after contrast administration to each other, thus obtaining the functional map and consequently the rrT1 of the whole liver at the voxel level; the second registration consisted of displaying the functional maps on the T1-weighted image 20 min after contrast administration, as seen in Fig. 3C.

3 $$MELIF \,score=0.694*(\frac{{height}_{p}^{0.6}}{{weight}_{p}^{0.3}*{liverVolume}^{0.6}})*100*(\sum_{x,y,z}^{liver}\frac{{T1}_{pre}\left(x,y,z\right)-{T1}_{postReg}(x,y,z)}{{T1}_{pre}(x,y,z)})$$

### Statistical analysis

GraphPad Prism 9.1.2 (GraphPad Software, LLC, San Diego, CA, USA) and Excel (Microsoft Office 2019, USA) were used for statistical analysis. Categorical variables are presented as absolute numbers and % values, and continuous variables are presented as medians (interquartile ranges (IQRs)) or means (± standard deviations). The D'Agostino and Pearson tests were used to test the data for normal and nonnormal distributions. The ALBI scores were converted to positive values to perform the test. All values were normally distributed, and only the MELD score was both not normally and not nonnormally distributed. The analysis of the CP scores was inconclusive, as they were not normally distributed, but the test for nonnormal distribution was not significant. As both the MELIF and the ALBI scores were normally distributed, parametric tests were used for the analysis. Pearson correlation was used and interpreted as follows: up to 0.1 as weak, 0.3 as moderate, and above 0.5 as strong. For pairwise comparison of the means, one-way ANOVA was performed. The Wilson/Brown method was used for receiver operating characteristic (ROC) analysis and calculation of the area under the ROC curve (AUC). The AUC results were interpreted according to the following classification: 0.9–1 = excellent, 0.8–0.9 = good, 0.7–0.8 = fair, 0.6–0.7 = poor and 0.5–0.6 = failed. The cut-off values were calculated using the Youden index^26^. In all analyses, a two-tailed p value less than 0.5 was considered significant.

## Data Availability

The manuscript contains all data supporting the findings of this study. The raw data used for this work are available upon reasonable request from the corresponding author: c.rio@skbs.de.
